# Decoding the Folding of *Burkholderia glumae* Lipase: Folding Intermediates *En Route* to Kinetic Stability

**DOI:** 10.1371/journal.pone.0036999

**Published:** 2012-05-15

**Authors:** Kris Pauwels, Manuel M. Sanchez del Pino, Georges Feller, Patrick Van Gelder

**Affiliations:** 1 Department of Structural Biology, VIB and Vrije Universiteit Brussel, Brussels, Belgium; 2 National Institute for Medical Research, Molecular Structure Division, London, United Kingdom; 3 Centro de Investigación Príncipe Felipe, Laboratorio de Proteómica, Valencia, Spain; 4 Laboratory of Biochemistry, Center for Protein Engineering, University of Liège, Liège-Sart Tilman, Belgium; 5 L-ProBE, Unit for Structural Biology, Ghent University, Ghent, Belgium; Russian Academy of Sciences, Institute for Biological Instrumentation, Russian Federation

## Abstract

The lipase produced by *Burkholderia glumae* folds spontaneously into an inactive near-native state and requires a periplasmic chaperone to reach its final active and secretion-competent fold. The *B. glumae* lipase-specific foldase (Lif) is classified as a member of the steric-chaperone family of which the propeptides of α-lytic protease and subtilisin are the best known representatives. Steric chaperones play a key role in conferring kinetic stability to proteins. However, until present there was no solid experimental evidence that Lif-dependent lipases are kinetically trapped enzymes. By combining thermal denaturation studies with proteolytic resistance experiments and the description of distinct folding intermediates, we demonstrate that the native lipase has a kinetically stable conformation. We show that a newly discovered molten globule-like conformation has distinct properties that clearly differ from those of the near-native intermediate state. The folding fingerprint of Lif-dependent lipases is put in the context of the protease-prodomain system and the comparison reveals clear differences that render the lipase-Lif systems unique. Limited proteolysis unveils structural differences between the near-native intermediate and the native conformation and sets the stage to shed light onto the nature of the kinetic barrier.

## Introduction

Several bacterial proteins, like the extracellular enzymes α-lytic protease and subtilisin, are very resistant to unfolding and proteolysis, although the thermodynamic stability of their native conformation compares to that of the unfolded state [1], [2]. A huge energetic barrier traps these proteins in a kinetically stable native state by preventing unfolding, but this implies a concurrent folding problem [3]. These proteins manage to fold spontaneously into an inactive, partially folded state and require the action of steric chaperones to lower the folding barrier by imprinting unique structural information to obtain their native and functionally active fold [4], [5].

*Burkholderia glumae*, an emerging phytopathogenic bacterium, produces such an enzyme via the type II secretion pathway [6], [7]. This protein, a lipase (LipA; EC 3.1.1.3), is first exported through the inner membrane with the concomitant removal of the signal sequence. A second translocation event through the outer membrane is mediated by a multiprotein assembly, called the Xcp-secreton, and is only possible after the indispensable periplasmic folding of such secreted proteins [8]. These lipases depend on a membrane-based, periplasmic steric chaperone, designated lipase-specific foldase (Lif), to obtain a soluble, biologically active and secretion-competent conformation [6], [9], [10]. The native state of the *B. glumae* lipase, which displays a globular α/β-hydrolase fold with a disulfide bond and a bound calcium ion, appears to be very stable as harsh conditions are required for its denaturation [11]–[13]. The significance of such stability is reflected in the industrial use of bacterial lipases as biocatalysts, whereby also (i) their hydrolytic proficiency, (ii) their ability to catalyze reactions under non-aqueous conditions, (iii) their well-known regio- and enantioselectivity and (iv) their application in the resolution of racemic mixtures to produce enantiopure compounds, makes them key players in biotechnological and pharmaceutical production processes [14]. In absence of Lif, denatured LipA can refold *in vitro* into a near-native intermediate conformation [15]. Addition of Lif to this inactive folding intermediate resulted in immediate activation and suggested the existence of a (un)folding barrier that prevents transient unfolding events and concomitant exposure to proteolysis.

The exact folding mechanism of bacterial lipases and the modus operandi of Lif remain enigmatic, since comprehensive understanding of the folding mechanism demands the in-depth characterization of partially (un)folded intermediates, transition states and their order in the folding process. Furthermore, *B. glumae* LipA was only suspected be to a kinetically trapped protein based on the fact that kinetic intermediates often accumulate when preceding a rate-limiting step in a folding pathway and based on similarities with secreted protease-prodomain systems whereby the protease folds into a molten globule in absence of its prodomain [2], [3], [5], [15]. However, direct evidence for its kinetic isolation was still lacking in the available literature. In the present work, thermally and chemically induced denaturation of the *B. glumae* LipA were used to characterize the native lipase conformation. The resulting folding fingerprint was complemented by comparison of the limited proteolysis of the native and near-native lipase conformations. A mass spectrometry analysis of the proteolytic fragments unveiled structural differences between the native and the near native state. Our findings therefore offer a platform to start understanding the structural changes that accompany the activation of the enzyme through Lif action.

## **Results**

### Thermal denaturation reveals a kinetically controlled system

**Figure 1:**
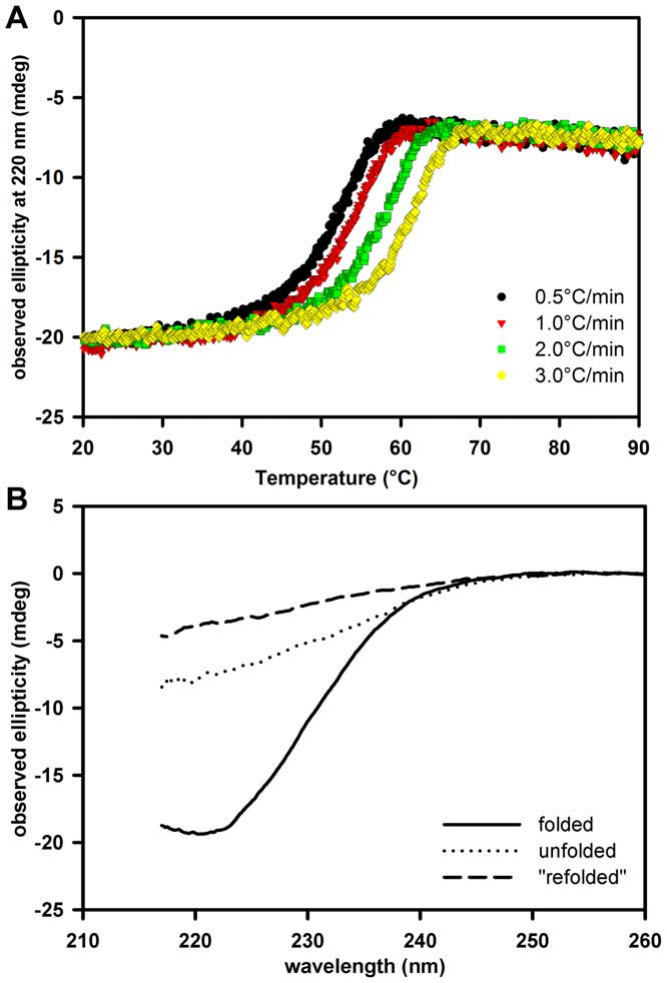
Scan rate dependent thermal denaturation of native *B. glumae* lipase monitored by far-UV CD spectroscopy. A) Thermal denaturation of *B. glumae* lipase in its native conformation (LipA_n_) in the presence of 6.6 M urea was measured at four different rates of heating. B) Wavelength scans of the folded state of LipA_n_ in the presence of urea at 20.0°C prior to thermal denaturation (solid line), the spectrum at 95.0°C (dotted line) and the spectrum of the lipase at 20.0°C after heating and cooling down (dashed line).

Thermal denaturation of native lipase (which we dubbed “LipA_n_”) was investigated via circular dichroism (CD) spectroscopy and differential scanning calorimetry (DSC). Consistent with the fact that LipA_n_ was shown to retain its fold in the presence of high concentrations of urea [13], we performed thermally induced unfolding using far-UV CD spectroscopy in the presence of 6.6 M urea in order to monitor a clear transition between the folded and unfolded signal (**Figure 1**). Under these experimental conditions (6.6 M urea, 20 mM NaPO_4_, 45 mM NaCl, pH 8.0) the application of heating rates varying from 0.5°C/min to 3.0°C/min resulted in an obvious variation of the melting point from 53.9°C to 62.5°C (**Figure 1A** **,** **Table 1**). The thermal transition was irreversible as evidenced by the far-UV wavelength spectra of the folded state prior to the heating and the spectrum measured upon cooling of the heated sample (**Figure 1B**).

**Table 1:** Scan rate dependence of the LipA_n_ conformation in the presence of 6.6 M urea as monitored by far-UV CD spectroscopy.

Scan rate (°C/min)	T_m_ (°C)
0.5	53.9
1.0	55.1
2.0	60.0
3.0	62.5

**Figure 2:**
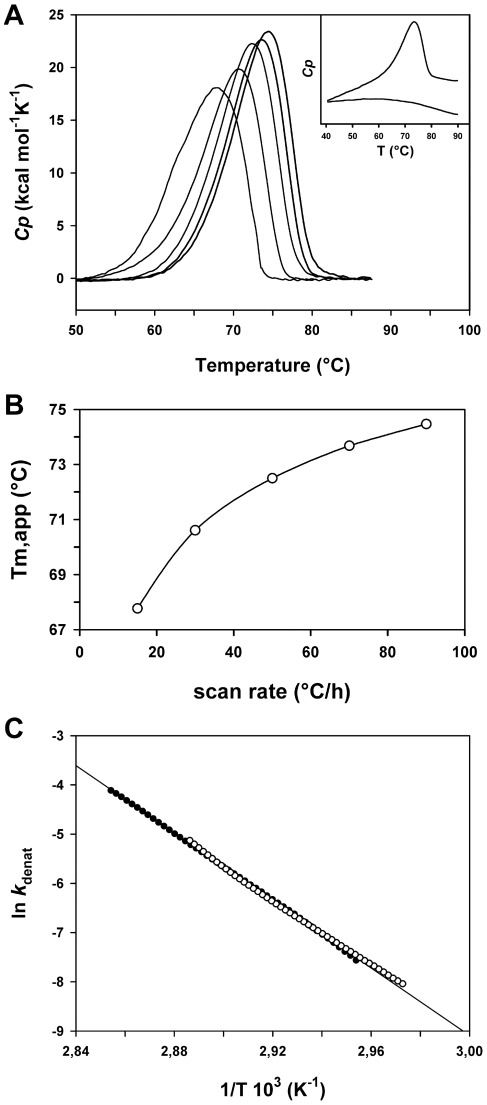
Differential scanning calorimetry analysis of LipA_n_. A) DSC thermograms of LipA_n_ unfolding in 30 mM MOPS, 1 M 3-(1-pyridinio)-1-propanesulfonate, pH 8.0. Thermograms are baseline subtracted and normalized for protein concentration. From left to right, scan-rates of 15, 30, 50, 70 and 90°C/h. Inset: raw DSC data of LipA_n_ at 90°C/h (upper curve) showing irreversibility upon rescanning (lower curve). B) Scan-rate dependence of the apparent melting point, *T_m,app_*, in irreversible unfolding of LipA_n_. C) Arrhenius plot for the temperature dependence of the denaturation kinetic constant, *k_denat_*. Data sets obtained at 30°C/h (open circles) and 90°C/h (closed circles).

In addition, we performed a DSC analysis of the thermal denaturation of LipA_n_ in the absence of chemical denaturants. When DSC experiments were performed in NaPO_4_ pH 8.0 as a buffer, the LipA_n_ aggregated during thermal denaturation (results not shown). Therefore, thermograms were recorded in MOPS buffer and in the presence of 3-(1-pyridinio)-1-propanesulfonate, a non-detergent sulfobetaine that prevents protein aggregation by both charge screening and hydrophobic screening effects [16], [17]. The DSC thermograms displayed a single slightly asymmetric peak (**Figure 2A**) and denaturation under these conditions was also completely irreversible as rescanning of a cooled sample after the first scan did not retrieve the initial signal (**Figure 2A** **, inset**). This indicates that irreversibility is an intrinsic property of LipA_n_ and is not a side effect of aggregation. As shown in **Figure 2A,B** the apparent melting point, *T_m,app_* was scan-rate dependent and shifted to higher values at fast scan rates, whereas the calorimetric enthalpy, *ΔH_cal_* (area below the transition) did not displayed significant variations (201±9 kcal mol^−1^). A similar behavior was recorded with or without the non-detergent sulfobetaine. This is the typical signature of an irreversible unfolding indicating that thermal denaturation of LipA_n_ is under kinetic control [18], [19]. The kinetically-driven unfolding of LipA_n_ was therefore analyzed according to a two-state irreversible model [18]:
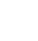
where N is the native state, I the irreversibly unfolded state and *k_denat_* a first-order kinetic constant, which changes with temperature according to the Arrhenius equation. This rate constant can be calculated by the relation:
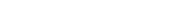
where *v* represents the scan rate (°C s^−1^), *Cp* the excess heat capacity at a given temperature, *ΔH*_cal_, the total heat of the unfolding process and *Q* the heat evolved at a given temperature [18]. The variation of *k_denat_* with temperature on an Arrhenius plot is illustrated in **Figure 2C**. For a true irreversible process, *k_denat_* is scan-rate independent, as indeed observed in the overlapping data sets obtained at 30 and 90°C in **Figure 2C**.

These complementary experiments show that the thermal unfolding of LipA_n_ is irreversible and that the melting points display scan-rate dependence in the presence and in the absence of chemical denaturants.

### Chemically induced denaturation reveals distinct LipA unfolding intermediates

**Figure 3:**
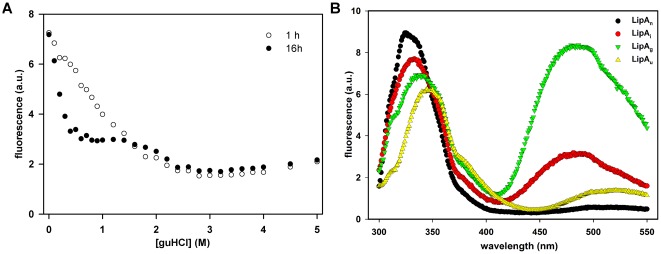
Identification of lipase folding intermediates through chemically induced denaturation using fluorescence. A) Chemically induced unfolding of native LipA from *B. glumae* is displayed by plotting the fluorescence emission intensity at 330 nm (in arbitrary units) against the denaturant concentration at two different incubation times (1 h incubation represented with open circles, 16 h incubation represented with black filled circles). GuHCl-induced denaturation of LipA_n_ upon 16 h incubation reveals the existence of an unfolding intermediate. B) Intrinsic protein fluorescence and ANS binding study for different lipase folding conformations: native lipase (LipA_n_; black circles), near-native intermediate (LipA_i_; red circles), molten globule-like conformation in 1.2 M guHCl (LipA_g_; green inverse triangles), unfolded lipase in presence of 6 M guHCl (LipA_u_; yellow triangles).

Since the existence of intermediates in the LipA folding landscape was already documented [15], we wanted to detect and characterize these intermediates through chemically induced denaturation of the native lipase. Indeed, El Khattabi and coworkers described a near-native folding intermediate, which we designate henceforth as “LipA_i_”, through chemically induced denaturation at elevated temperature and subsequent rapid refolding. We resorted to addition of guHCl to LipA_n_, which resulted in an immediate loss of enzymatic activity and a red-shift of the intrinsic fluorescence maximum. Interestingly, the denaturation curve recorded after 16 h of incubation revealed the existence of a stable folding intermediate (referred to as LipA_g_), which appeared in the 1.0–1.4 M guHCl range (**Figure 3A**). LipA_g_ is characterized by an intrinsic fluorescence emission maximum (λ_em,max_) at 338 nm and exhibits a substantial interaction with ANS. This clearly differs from the spectral properties of native lipase, which exhibits a λ_em,max_ at 325 nm, and fully unfolded lipase (LipA_u_) that has a λ_em,max_ at 350 nm. Both LipA_n_ and LipA_u_ do not bind ANS (**Figure 3B**).

Since refolded LipA is known to adopt an inactive near-native conformation, we followed the protocol described by El Khattabi and coworkers [15] and characterized its fluorescence properties to compare them with our present findings of LipA_g_. LipA_i_ has a λ_em,max_ at 332 nm and binds modestly to ANS in comparison to LipA_g_ (**Figure 3B**). The λ_em,max_ of LipA_i_ is red-shifted as compared to LipA_n_ and suggests that the tryptophans are still in a mainly non-polar environment.

Finally, we applied size exclusion chromatography to the different LipA conformations to gain insight on the hydrodynamic properties of the observed species (see figure S1). LipA_n_ eluted as a single peak with an apparent Mw of 28 kDa, which is slightly lower than the expected 33 kDa and confirms its compact monomeric nature. LipA_i_ elutes at an apparent Mw of 33 kDa, indicating that LipA_i_ adopts a slightly more expanded shape than LipA_n_. LipA_g_ elutes with an apparent Mw of 53 kDa corresponding to a more expanded conformation when compared to LipA_i_ and LipA_n_, while LipA_u_ elutes as a 158 kDa protein.

Thus, a combination of intrinsic fluorescence, ANS binding and gelfiltration allowed us to discriminate between the two distinct lipase intermediates and to contrast these with the native and fully unfolded lipase conformations.

### Lif does not bind the molten globule-like conformation of LipA

**Figure 4:**
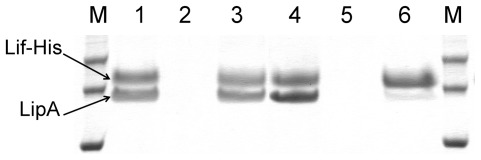
Interaction chromatography of LipA-Lif to probe binding in presence and absence of guHCl. Specific retention of LipA that is applied on a Ni-NTA column with immobilized Lif, in absence (lanes 1–3) or presence (lanes 4–6) of 1.2 M guHCl. Lanes 1 and 4 represent the flow through fraction, lanes 2 and 5 represent the wash fraction and lanes 3 and 6 contain the proteins that elute from the column. M indicates the molecular weight marker of which the 45 kDa, 35 kDa and 25 kDa bands are represented.

In agreement with the previous report [15], we found that LipA_i_ is indeed activated upon Lif addition, confirming its nature as a true folding intermediate (results not shown). Therefore we wanted to know if Lif can also bind LipA_g_. Interaction chromatography gives direct evidence that LipA and its cognate His-tagged Lif do not interact in the presence of 1.2 M guHCl (**Figure 4**). When the LipA-Lif complex was applied to a Ni-loaded affinity column under native conditions (i.e. 100 mM Tris-HCl pH 8.0), the complex was recovered in the eluted fraction. However, when the complex was pre-incubated in 1.2 M guHCl, only the His-tagged Lif was retained on the column and recovered after elution, whereas the lipase only appeared in the void fraction.

### Limited proteolysis of native and near-native intermediate lipase

**Figure 5:**
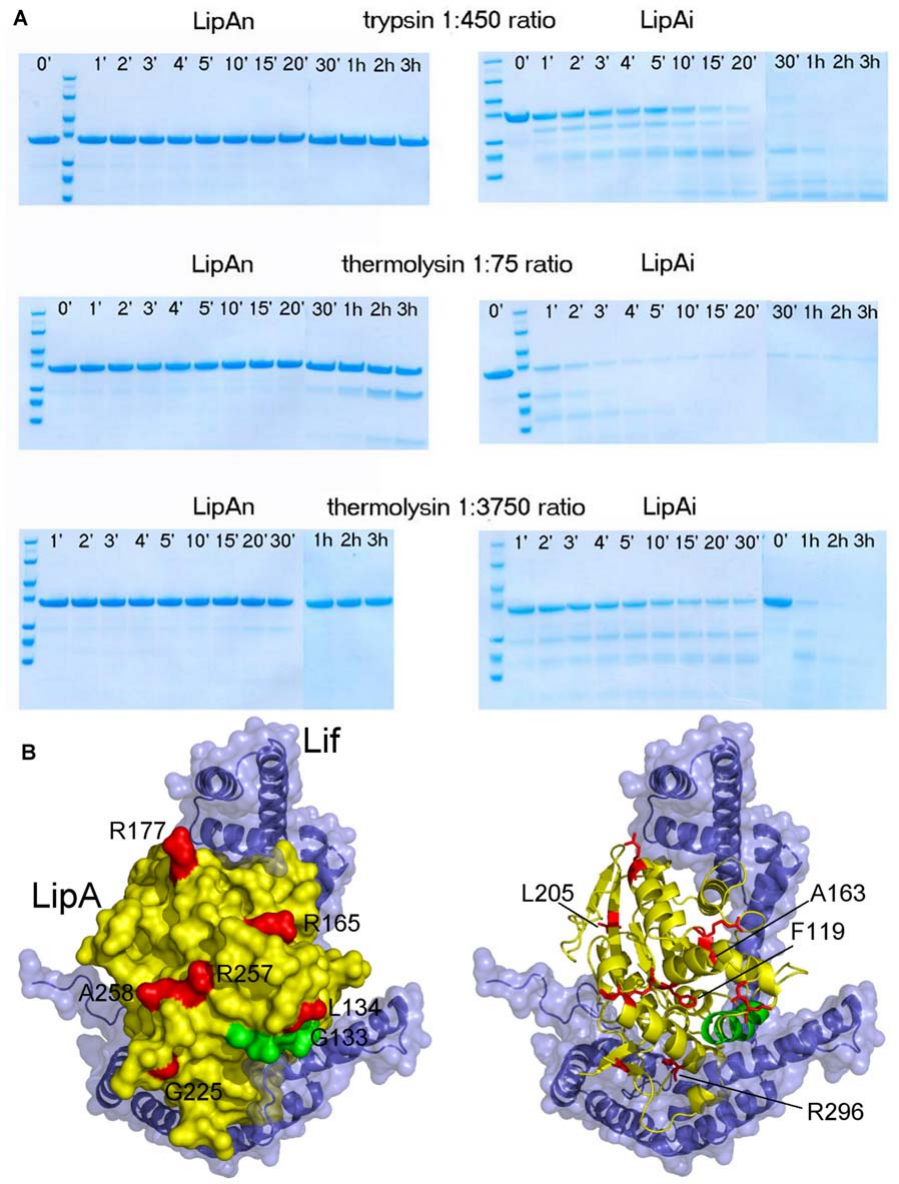
Limited proteolysis of the native and near-native conformation of *B. glumae* LipA. A) Time-derived limited proteolysis of the native lipase (LipA_n_, left panels) in comparison to the near-native conformation (LipA_i_, right panels) using thermolysin and trypsin in the protease∶LipA ratios as indicated. The marker proteins from top to bottom have respective Mw of 116.0 kDa, 66.2 kDa, 45.0 kDa, 35.0 kDa, 25.0 kDa, 18.4 kDa and 14.4 kDa. The time points at which samples were taken are indicated above each lane. B) Structural representation of the limited proteolytic sites in *B. glumae* LipA_n_. The limited proteolysis results are interpreted in the context of the lipase-Lif interaction and the figure was prepared using PDB entry 2ES4 and the visualization program PYMOL [34]. The globular lipase is represented in yellow and the movable lid that covers the lipase active site (α-helix 5) is highlighted in green. The embracing Lif is represented in blue in cartoon with a semitransparent surface. The hot spots of limited proteolysis are represented in red, on the surface representation of LipA in the left panel and in the red sticks representation in the right panel. While F119 and L205 are not solvent exposed and indicated on the right panel, A163 is not visible on the LipA surface in the left panel due to the orientation of the molecule and therefore also indicated on the right panel.

The intermediate fold of LipA is near-native, but enzymatically inactive [15]. To probe the differences between the native and intermediate form, we have performed limited proteolysis experiments. Limited proteolysis typically occurs in flexible loop regions that are solvent exposed and devoid of regular secondary structure or that are prone to local unfolding [20]. Although this dogma was recently challenged [21], the structure and dynamics of the substrate protein play a crucial role in limiting the proteolysis, particularly when comparing different conformations of the same protein [22]. We used trypsin and thermolysin to perform a controlled proteolytic digestion and analyzed different reaction times. The Coomassie-stained gels provide a picture of the time course and the extent of cleavage for the partial proteolytic digestion of native lipase (LipA_n_) and the near native intermediate lipase (LipA_i_) (**Figure 5A**).

As observed in the left panel of **Figure 5A**, LipA_n_ was completely resistant to proteolysis by trypsin or thermolysin. LipA_i_ on the other hand was completely degraded within 2 hours under identical experimental conditions. Cleavage of LipA_i_ revealed two dominant bands of ∼30 kDa and ∼18 kDa and of ∼25 kDa and ∼18 kDa for trypsin and thermolysin, respectively, thereby indicating that certain cleavage sites are preferred. Only a high amount of thermolysin (in a 1∶75 thermolysin∶LipA_n_ (*w/w*) ratio) gave rise to a proteolytic fragment of ∼25 kDa, which only became visible after 1 h incubation. In contrast, LipA_i_ was completely degraded within 5 min under identical conditions with such high amount of thermolysin.

Several predominant protein bands from the Coomassie-stained SDSPAGE were excised and subjected to MS analysis. The results of the peptidic fragment characterization are displayed in **Table 2**. All thermolysin and tryptic cleavage sites are located at the solvent accessible surface of the protein and mostly in loops and turns or at the termini of secondary structure elements (**Figure 5B**). Only thermolysin cleavages at L205 and F119 form an exception to this observation since they are buried and located in β-strand β6 and at the N-terminus of helix α4, respectively.

**Table 2:** Limited proteolysis fragments of LipA_i_ that were detected through MS analysis.

Protease	Residues Nt to Ct	MW of fragments (kDa)
**Thermolysin**	1–319	33.1
	1–258	26.4
	1–205	21.1
	134–319	19.1
	1–163	17.0
	1–133	14.0
	5–119	12.1
**Trypsin**	1–296	30.7
	1–257	26.4
	1–225	23.2
	1–177	18.6
	1–165	17.3

The sequence covered by MS and their theoretical mass are indicated.

Although both proteases have a different specificity, our results show that proteolysis coincides in two places: A163/R165 and R257/A258. The R257/A258 “hot spot” for cleavage is located directly upstream in the sequence and structure from the catalytic residue D263 and at the C-terminus of helix α9, while A163/R165 are located at the C-terminus of helix α6 [10], [12].

The tryptic cleavage at R296 is adjacent to V295, which is conserved in the calcium binding site. F119 and L134 are located at the boundaries of helix α4, while R177 is situated at the C-terminus of helix α7 and G225 is found in a mobile loop of the β-hairpin motif in the LipA structure. Notably, helices α4 and α6 flank the amphipathic helix α5 (**Figure 5B**, highlighted in green), which is known as the movable lid that covers the active site [12].

## **Discussion**

Folding intermediates and kinetically trapped states are often observed when intrinsically slow reactions are associated with the folding process [23]. In *B. glumae* lipase the decisive folding step is catalyzed by Lif, since in the absence of this steric chaperone, LipA cannot fold autonomously into its biologically active and secretion-competent conformation within a physiologically relevant timeframe [6], [15]. This is similar to subtilisin and α-lytic proteases where the final active conformation is entirely dependent on the action of the propeptide [2]. Interestingly, the guHCl-induced denaturation profile of native LipA resembles earlier unfolding studies on pro-subtilisin and a subtilisin intermediate for which also two transitions were detected [24]. A combination of Trp fluorescence, ANS binding and size exclusion chromatography led us to conclude that a newly discovered intermediate, LipA_g_ that exists in the 1.0–1.4 M guHCl-window, agrees with the operational definition of a molten globule [25]–[27]. However, this molten globule-like conformation is clearly distinct from the near-native intermediate (LipA_i_) and was only observed under artificial conditions (i.e. in the presence of moderate quantities of guanidine HCl). There are no *in vivo* observations for the existence of LipA_g_ and, in addition, a column retention assay using affinity chromatography confirmed that Lif does not bind to LipA_g_. Although the expanded hydrodynamic radius of LipA_g_, in contrast to the compact shape of LipA_n_ and LipA_i_, might directly prevent the interaction with Lif, it can also be conceived that the presence of guHCl interferes with the H-bonding network that stabilizes the protein-protein interaction interface [10]. On the other hand, Lif could also be partially denatured in the presence of 1.2 M guHCl and as such be hampered in binding LipA_g_. However, from denaturation experiments we know that the midpoint of denaturation for Lif is at 2.2 M guHCl at 25°C and that the Lif fold is likely still intact (unpublished observations).

Another parallelism between prodomain-dependent proteases and the lipase is based on scan-rate dependent thermal denaturation data. By using complementary techniques to probe the thermal unfolding of LipA_n_, a clear dependence of the T_m_ values with the heating rate was observed. This provided unambiguous evidence that LipA_n_ is indeed a kinetically controlled conformation, like the proteases reliant on prodomains for their biogenesis. As such it is experimentally confirmed that the lipase stability arises from the kinetic barrier that blocks the native conformation from unfolding, rather than from equilibrium thermodynamics. Together with its high resistance to proteolysis (**Figure 5**) this explains the longevity of the secreted enzyme in hostile environment in which it has to operate [28].

Nonetheless, our unfolding studies of the *B. glumae* lipase yielded an intriguing folding fingerprint that showcases at least two well-spaced intermediates in the folding landscape. This is strikingly different from the well-studied α-lytic protease and subtilisin systems where the unfolded polypeptide folds into a molten globule with hardly any tertiary structure formation [3]. Instead, our studies strongly suggest that lipase can fold further along the folding pathway into a near-native state. Taken together, our studies suggest that Lif and its cognate lipase represent a novel system that accommodates complex folding profiles.

Understanding the nature of the folding barrier will require a detailed comparative study of the structures of LipA_i_ and LipA_n_. Since LipA_i_ is vulnerable to proteolytic attack and gave rise to several discrete peptidic fragments, in sharp contrast to LipA_n_, a combination of limited proteolysis and MS analysis lead to the successful identification of the fragments and the initial cleavage sites in LipA_i_ (**Figure 5** **,** **Table 2**). While all cleavage sites are located at the solvent accessible surface of the protein, L205 and F119 are buried in the hydrophobic core and located within a β-strand and α-helix, respectively. It is likely that the two fragments that lead to their identification originate from secondary cleavage events by thermolysin, where an initial proteolytic event would allow the (partial) unfolding of those secondary structure elements and pave the way for additional proteolysis of the protein. Curiously, all major proteolytic cleavage sites in LipA_i_ are found opposite to the contact area with Lif [10] (**Figure 5B**). Apparently the acquired protease resistance of LipA_n_ is not due to a direct contact with the Lif but might be obtained via a remote conformational rearrangement of the loop regions around helix α4, helix α6, helix α9 (with a positioning of the active site residue D263) and around β-strand β6. Moreover, the detection of the regions where Lif influences the rearrangement to the proteolytic resistant conformation can also help to advance our understanding of the type II secretion motif, because LipA_i_ remains in the periplasm while LipA_n_ gets transported to the extracellular medium [6], [29].

**Figure 6:**
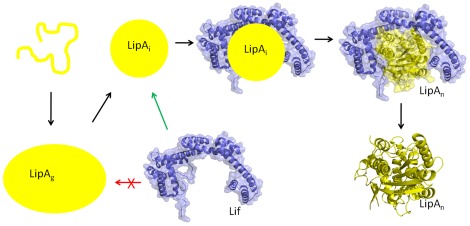
Proposed hypothetical folding model for the *B. glumae* lipase based on our observations. We suggest that the biogenesis of lipase encompasses several steps: (i) after translation, the lipase is translocated over the inner membrane in a Sec-dependent and therefore unfolded conformation step with the concomitant removal of the leader peptide; (ii) LipA folds through a short-lived transient molten globule-like state (LipA_g_) that was observed under moderate guHCl concentrations into a more compact and near native folding intermediate (LipA_i_); (iii) rather than LipA_g_, this near-native intermediate LipA_i_ is the substrate that specifically interacts with Lif and (iv) becomes fully folded and activated into the native LipA_n_ fold through a contact-assisted folding mechanism. Several questions remain to be addressed regarding the disulfide bond formation, the incorporation of the calcium ion, how LipA_n_ is released from Lif, as well as the chronology of these events. This hypothetical model is only based on *in vitro* observations and as detailed thermodynamic and kinetic data remain elusive, the arrows only represent the subsequent transitions in the lipase folding landscape, while they do not enclose any absolute kinetic or equilibrium information.

In conclusion, we propose a model for lipase folding whereby the spontaneous folding of LipA might proceed via a transient molten globule-state, LipA_g_, into a compact, and stably populated near-native conformation (**Figure 6**). LipA_i_ is likely to be located downhill along the folding pathway and would therefore be structurally closer to the native conformation. In this regard, Lif would only recognize and bind LipA_i_, thereby protecting hydrophobic patches in LipA_i_ that leads to a more compact, rigid and protease resistant structure. In this context, it is also appropriate to mention that it was already speculated that the presence of water molecules might stabilize LipA_i_ and that Lif would lower the energy barrier through removal of those waters [15]. More recently, it was proposed that a solvation barrier would contribute to the kinetic stability of the fungal lipase of *Thermomyces lanuginosus* [30]. Our results would fit a folding mechanism in which most of the structural formation of the protein is achieved spontaneously, whereby Lif expels water from the hydrophobic patches and cements the LipA in its native and biologically active conformation through propagation of binding interactions to remote sites within LipA. However, this putative mechanism requires more experimental evidence and particularly insights in (un)folding kinetics of the lipase. Our observations set the stage for profound mutagenesis and kinetic studies that should further probe the differences between the native and intermediate forms. In combination with the available crystal structures of LipA, this information will lead to an advanced understanding of the kinetic isolation of the native lipase through Lif mediation.

## Materials and Methods

### Materials

*B. glumae* LipA and Lif were produced and purified as described previously [31], [32]. Urea (>99.8% purity) was purchased from Rose Chemicals, guHCl (>99.5% purity) was obtained from Fluka, Tris (PlusOne) from Pharmacia Biotech. Sequencing grade trypsin and GluC were purchased from Promega and Sigma, respectively and thermolysin was obtained from Calbiochem. Trifluoroacetic acid (TFA), acetonitrile (ACN), 3-morpholinopropane-1-sulfonic acid (MOPS), 1-anilino 8-naphtalene sulfonate (ANS) and Coomassie Brilliant Blue R250 were purchased from Sigma.

### Preparation of the near-native intermediate folding conformation LipA_i_

The near-native intermediate LipA conformation (LipA_i_) was prepared based on the protocol of El Khattabi *et al.* [15]. First, native lipase (LipA_n_) was dialyzed against milliQ water (Slide-A-Lyzer dialysis unit, Pierce), followed by a speedvac step. The resulting pellet was resuspended in 8 M urea, 50 mM Tris-HCl pH 8.0, 12 mM EDTA and incubated at 56°C for 1 h. Refolding to LipA_i_ was induced by a 100-fold rapid dilution in 100 mM Tris-HCl pH 8.0 at room temperature.

### Analytical size-exclusion chromatography

Analytical size-exclusion chromatography was performed at room temperature using a Superdex-75 HR 10/30 column (Amersham Bioscience) equilibrated with 20 mM Na phosphate, 45 mM NaCl, 0.6 mM EDTA, 90 mM urea, pH 7.0. Gelfiltrations for LipA_g_ (molten globule) and LipA_u_ (unfolded conformation) were performed in the buffer supplemented with 1.2 M and 6 M guHCl, respectively. Protein samples of 50 µg and at a concentration of 0.5 mg/mL were loaded on the column using an Äkta basic HPLC system at a flow rate of 0.5 mL/min. The column was calibrated with γ-Globulin (158 kDa), ovalbumin (44 kDa), myoglobin (17 kDa) and vitamin B12 (1.35 kDa), to estimate the apparent molecular weight of the proteins in the elution peaks.

### Circular dichroism spectroscopy

CD data were recorded on a J-715 spectropolarimeter (JASCO) equipped with a cell holder thermostatted by a PTC 348-WI Peltier unit. Thermal denaturation curves were recorded with 5 µM lipase in 20 mM NaPO_4_, 6.6 M urea, 150 mM NaCl, pH 7.8 with a 0.1 cm path length quartz cuvette (Hellma). The change in the CD-signal intensity at 220 nm was monitored at 0.1°C intervals from 20 to 95°C while increasing the temperature at varying rates (in the range of 0.5°C/min to 3.0°C/min). The melting points (midpoint of transition) were obtained by calculating the first derivative of the experimental curve.

### Fluorescence spectroscopy

Fluorescence emission spectra were recorded using an AMINCO-Bowman Series2 luminescence spectrometer (Spectronic Instruments) at 25°C with excitation at 280 nm. The cell holder was thermally controlled using a water bath to maintain the temperature of the sample. The slit width of both monochromators was 4 nm. Chemically induced denaturation was followed by measuring the changes in intrinsic fluorescence emission between 300 and 370 nm at guHCl concentrations of 0–6 M. The samples each containing 0.5 µM lipase were prepared using a Hamilton MDL 503B serial dispenser by combining stock solutions of 6 M guHCl in 100 mM Tris-HCl (pH 8.0) with 100 mM Tris-HCl pH 8.0 to the appropriate denaturant concentration.

ANS binding was monitored with excitation at 280 nm and emission spectra were scanned in the range of 300 to 550 nm with a 4 nm bandpass at a speed of 1 nm/min. A final concentration of 50 µM ANS was added to 1.2 µM lipase and the mixture was incubated for 1 h at 25°C prior to measurement.

### Differential scanning calorimetry

All calorimetric experiments were performed using a MicroCal VP-DSC differential scanning microcalorimeter with a 0.515 ml sample cell, under ∼25 psi positive cell pressure, at scan rates of 15, 30, 50, 70 and 90°C h^−1^ and at ∼0.5 mg/ml (15 µM) protein concentration in the sample cell. LipA_n_ samples were dialyzed overnight against 30 mM MOPS, pH 8.0. Before experiments, an equal volume of 2 M 3-(1-pyridinio)-1-propanesulfonate prepared in the dialysis buffer was added to both the protein sample and the reference buffer [16]. The instrumental baseline was determined with both cells filled with the reference buffer. Reversibility of thermally induced denaturation was checked by reheating the solution after cooling from the previous upscan. All experiments were repeated at least once to guarantee reproducibility. The DSC data were analyzed after subtraction of the instrumental baseline with the Microcal Origin DSC v.7.0 software package. Calorimetric enthalpies (*ΔH_cal_*) were determined as the area of the transitions, normalized for protein concentration and limited by a progress baseline or by a cubic connect.

### IMAC column retention

To test the interaction of LipA and Lif, 30 µg LipA-Lif complex was prepared in a total volume of 400 µl 100 mM Tris-HCl pH 8.0 supplemented with 1.2 M guHCl (the positive control contained no guHCl). After 16 h incubation at 25°C, the mixture was applied to a NiNTA Spin column (Qiagen) that was equilibrated with the appropriate buffer and processed according to the manufacturer's recommendations. The ‘flow through’ fraction was collected and the column was washed with 400 µl of the respective buffer, yielding the ‘wash’ fraction. Bound proteins were eluted by the appropriate buffer supplemented with 400 mM imidazole. All samples were subjected to a trichloroacetic acid (TCA) precipitation prior to SDS-PAGE analysis.

### Limited proteolysis

For partial digestion of native lipase, 25 µl of *B. glumae* LipA solution (17.7 µg/µl in 0.1 M Tris-HCl pH 8.0) was speed-vacced and redissolved in 10 µl 100 mM NaPO_4_ pH 7.5 followed by 30 min incubation at 25°C. Next, 1 ml of refolding buffer (10 mM Tris-HCl, 5 mM CaCl_2_, pH 8.0) was added. To 250 µl of this sample either 0.25 µg trypsin (2.5 µl of 0.1 µg/µl), 1.5 µg thermolysin (2.5 µl of 0.6 µg/µl) or 30 ng thermolysin (2.5 µl of 12 ng/µl) was added, briefly vortexed and incubated at 25°C in a waterbath. Samples of 20 µl were taken at different time points, and the proteolysis reaction was quenched immediately through addition of EDTA, leupeptin and AEBSF. After 15 min incubation on ice, protein loading buffer was added and samples were heated at 95°C for 5 min prior to SDS-PAGE.

For partial digestion of LipA_i_, the lipase pellet after speedvaccing was redissolved in 10 µl 100 mM NaPO_4_ pH 7.5, 14 mM EDTA, 9 M urea and incubated for 1 h at 56°C. Next, the unfolded lipase was refolded in 1 ml refolding buffer (10 mM Tris-HCl, 5 mM CaCl_2_, pH 8.0) and processed identically as the native lipase.

For SDS-PAGE and MS analysis of the limited proteolysis, 1 mm thick NuPAGE Novex 10% Bis-Tris precast gels (Invitrogen) were used with freshly prepared MES-buffer. The gels were stained with freshly made Coomassie Brilliant Blue solution (0.1% CBB R250, 50% methanol, 10% acetic acid) and extensive destaining was performed to efficiently remove the SDS. Finally, the gels were put in bidistilled water.

### Mass Spectrometry analysis

The gel bands were excised conservatively and digested as described elsewhere [33], using trypsin or GluC for samples previously digested with thermolysin or trypsin, respectively. The resulting peptides were dried in a vacuum centrifuge, resuspended in 7 µL of 0.1% TFA, and 1 µL was spotted onto the MALDI target plate. After the droplets were air-dried at room temperature, 0.5 µL of matrix (5 mg/mL CHCA (α-cyano-4-hydroxycinnamic acid, Sigma) in 0.1% TFA-ACN/H2O (1∶1, *v/v*) was added and air-dried at room temperature. The resulting samples were analyzed in a 4700 Proteomics Analyzer (Applied Biosystems, Foster City, USA) in positive reflectron mode (2000 shots every position). Five of the most intense precursors (according to the threshold criteria: minimum signal-to-noise: 10, minimum cluster area: 500, maximum precursor gap: 200 ppm, maximum fraction gap: 4) were selected for every position for the MSMS analysis. MS/MS data was acquired using the default 1 kV MS/MS method. External calibration of the MALDI TOF instrument was performed using the 4700 Cal Mix (Applied Biosystems) according to the manufacturer's indications. For MS/MS calibration, the fragmentation of Angiotensin I included in the 4700 Cal Mix was used. Alternatively, the peptide mixture was analyzed by LC-MS/MS using an Ultimate nano-LC system (LC Packings) and a QSTAR XL Q-TOF hybrid mass spectrometer (MDS Sciex, Applied Biosystems, Concord, Canada). Samples (5 µl) were delivered to the system using a FAMOS autosampler (LC Packings) at 30 µl/min, and the peptides were trapped onto a PepMap C18 precolumn (5 mm 300 m i.d.; LC Packings). Peptides were then eluted onto the PepMap C18 analytical column (15 cm 75 m i.d.; LC Packings) at 200 nl/min and separated using a 55 min gradient of 15%–35% ACN. The QSTAR XL was operated in an information-dependent acquisition mode. Acquisitions of a 1-s TOF MS scans from 400 to 2000 m/z were followed by 3-s product ion scans from 65 to 2000 m/z of the three most intense doubly or triply charged ions. The QSTAR-XL TOF was calibrated with a mixture of CsI and cPDI inhibitor.

A local database containing the lipase sequence was searched with MASCOT (Matrix-Science). The MS and MS/MS information was sent to MASCOT via the GPS software (Applied Biosystems) or Mascot Daemon depending on the instrument. Searches were done with tryptic or GluC specificity allowing one missed cleavage or with no enzyme. The mass tolerance was set to 100 ppm in MS mode and 0.8 Da for MS/MS data. Carbamidomethylation of Cys was used as a fixed modification and oxidation of Met and deamidation of Asn and Gln as variable modifications.

## Supporting Information

Figure S1 **Analytical size exclusion chromatography of the different lipase conformations.** The hydrodynamic properties of the different lipase conformations were investigated by analytical gelfiltration chromatography (Superdex-75 HR10/30TM). The partition coefficient, K_av_, which is a measure of the elution behavior, was calculated based on the equation K_av_ = (V_e_−V_o_)/(V_t_−V_o_) with V_e_ being the elution volume, V_t_ the total volume of the column (23.56 mL) and V_o_ the void volume of the column as determined by dextran blue (5.9 mL). γ-Globulin (158 kDa), ovalbumin (44 kDa), myoglobin (17 kDa) and vitamin B12 (1.35 kDa) were used as calibration standards to derive the apparent molecular weights of the LipA conformations.
(DOCX)
