# Supplementary material for: Decoding the Folding of Burkholderia glumae Lipase: Folding Intermediates En Route to Kinetic Stability
Source: PLoS One. 2012 May 15;7(5):e36999. doi: 10.1371/journal.pone.0036999 (PMC3352829; doi:10.1371/journal.pone.0036999)

**Supplementary figure**

**Figure S1. Analytical size exclusion chromatography of the different lipase conformations.** The hydrodynamic properties of the different lipase conformations were investigated by analytical gelfiltration chromatography (Superdex-75 HR10/30TM). The partition coefficient, K_av_, which is a measure of the elution behaviour, was calculated based on the equation K_av_=(V_e_-V_o_)/(V_t_-V_o_) with V_e_ being the elution volume, V_t_ the total volume of the column (23.56 mL) and V_o_ the void volume of the column as determined by dextran blue (5.9 mL). γ-Globulin (158 kDa), ovalbumin (44 kDa), myoglobin (17 kDa) and vitamin B12 (1.35 kDa) were used as calibration standards to derive the apparent molecular weights of the LipA conformations.


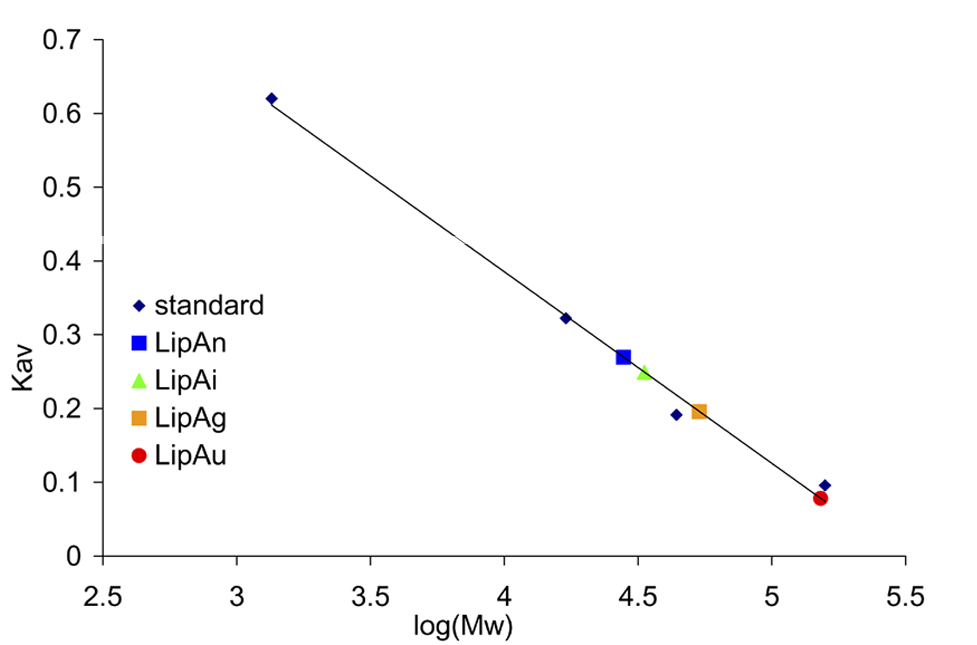

Supplement: Figure S1 — Analytical size exclusion chromatography of the different lipase conformations. The hydrodynamic properties of the different lipase conformations were investigated by analytical gelfiltration chromatography (Superdex-75 HR10/30TM). The partition coefficient, Kav, which is a measure of the elution behavior, was calculated based on the equation Kav = (Ve−Vo)/(Vt−Vo) with Ve being the elution volume, Vt the total volume of the column (23.56 mL) and Vo the void volume of the column as determined by dextran blue (5.9 mL). γ-Globulin (158 kDa), ovalbumin (44 kDa), myoglobin (17 kDa) and vitamin B12 (1.35 kDa) were used as calibration standards to derive the apparent molecular weights of the LipA conformations. (DOCX) [file pone.0036999.s001.docx]
